# Editorial: Structural biology: a gateway to understanding metabolic and signaling pathways in plants

**DOI:** 10.3389/fpls.2025.1773533

**Published:** 2026-01-20

**Authors:** Isabel Nogués, Bartosz Sekula, Milosz Ruszkowski

**Affiliations:** 1 Institute of Research on Terrestrial Ecosystems, National Research Council, Rome, Italy; 2 Institute of Molecular and Industrial Biotechnology, Lodz University of Technology, Lodz, Poland; 3 Department of Structural Biology of Eukaryotes, Institute of Bioorganic Chemistry, Polish Academy of Sciences, Poznan, Poland

**Keywords:** cytokinin signaling pathway, herbicide design, histidine biosynthesis pathway, histidine-containing phosphotransfer protein, ornithine transcarbamylase, polyglutamylated tetrahydrofolate, serine hydroxymethyltransferase

Understanding plant protein structure offers concrete insight into how these fascinating organisms perceive and respond to environmental and hormonal cues, regulate metabolic pathways, and adapt to stress. Structural analyses reveal how proteins interact and undergo conformational shifts, events that underpin signal transduction, stress tolerance, and nutrient metabolism. Structural data also clarifies enzyme function and specificity, which are essential for designing molecules that boost plant resilience or block certain pathways. Such data directly support the development of targeted herbicides, anti-metabolite compounds, and improved strategies for enhancing crop resistance to pathogens and environmental. Additionally, comparing protein structures across species reveals evolutionary adaptations and functionally important regions.

Owuocha et al. reported the first high-resolution (1.7 Å) crystal structure of soybean serine hydroxymethyltransferase 8 (SHMT8) bound to a diglutamylated form of tetrahydrofolate (THF), a key cofactor in one-carbon metabolism. Although THF polyglutamylation had long been known to be essential for folate homeostasis and to enhance enzyme affinity, the structural basis for these effects had remained unclear. The SHMT8 complex with diglutamylated THF revealed a marked rearrangement of the THF-binding loop comprising residues 377 to 385, and the most prominent change was a rotation of Leu383 that permitted the formation of new hydrogen bonds with the two glutamate residues of the ligand. These contacts stabilized the enzyme and cofactor complex, providing a structural explanation for the increased affinity associated with longer polyglutamate chains. Thermal shift assays and spectroscopic measurements supported this interpretation by showing greater enzyme stability and tighter folate binding as the tail length increased. Taken together, these findings demonstrated how SHMT8 relies on loop flexibility to recognize polyglutamylated folates, a major regulatory feature in plant metabolism, and they also provided a structural foundation for the design of antifolate inhibitors that could exploit loop closure and electrostatic complementarity. The study therefore linked specific conformational changes to functional outcomes, advanced understanding of folate-dependent pathways, and supplied a framework for the development of herbicides and therapeutic agents.

While two-component signaling is widespread in bacteria, plants employ a more elaborate variant, the multistep phosphorelay (MSP). It proceeds through four consecutive phosphorylation events at the kinase’s catalytic histidine, its receiver domain, a histidine-containing phosphotransfer protein (AHP), and finally a response regulator (ARR). MSP underlays the action of cytokinin and ethylene, two hormones central to plant biology. Yet, the structural basis of this cascade remains incomplete, which limits mechanistic insight into how plants integrate signals from internal and external stimuli. Tran and Ruszkowski helped narrow this gap by solving the structure of the *Arabidopsis thaliana* AHP1 and ARR1 complex. ARR1 contains a GARP DNA-binding domain and a canonical receiver domain that contacts AHP1. The structure supported a model in which the receiver domain represses the GARP domain, with MSP-driven phosphorylation of the receiver domain likely relieving this inhibition. Biolayer interferometry further showed that even without phosphotransfer ARR1 is capable of binding AHP1 only about twice as strongly as AHP2-AHP5, which implied that even unphosphorylated AHPs might still interact with ARR1 and potentially sequester it.

Structural insight is essential for rational inhibitor design, including the development of new herbicides in applied plant biology. One promising target is the histidine biosynthesis pathway, particularly imidazole-glycerol phosphate dehydratase (IGPD, EC 4.2.1.19), known as HISN5 in plants. Witek et al. analyzed HISN5 from the model legume *Medicago truncatula* using both X-ray crystallography and cryogenic electron microscopy, achieving a cryoEM map at 2.2 Å resolution that allowed for a detailed examination of enzyme-inhibitor interactions. Their work identified ligand binding hotspots and included a virtual screening campaign that uncovered candidate molecules and linkers for merging fragments. Because reliable activity assays are crucial for evaluating inhibitor candidates, they also introduced a new method for measuring HISN5 activity based on isothermal titration calorimetry combined with enzymatic synthesis of its substrate.

The study by Nielipinski et al. demonstrated that strategies found in nature can serve as inspiration for inhibitor design, as pathogenic microorganisms utilize their metabolites to inhibit key plant enzymes and metabolic pathways, thereby promoting invasion. The authors determined the crystal structures of *Arabidopsis thaliana* ornithine transcarbamylase (OTC), which revealed the binding modes of its substrates, ornithine and carbamoyl phosphate, and examined OTC inhibition by phaseolotoxin, an antimetabolite produced by *Pseudomonas syringae* pathovars. The toxin is metabolized by enzymatic machinery in the invaded plant to release octicidine, which acts as an irreversible and competitive OTC inhibitor, which causes severe disease in several crop species. Structural data revealed that the enzyme underwent a pronounced open-to-closed transition of the active site loop upon substrate binding, involving loop bending and partial helix unwinding. This transition explained why phaseolotoxin was a weaker OTC inhibitor than octicidine. While octicidine mimicked the reaction transition state and locked the active site lid in a closed conformation, the intact toxin could not bind efficiently because of possible steric hindrance.

These studies collectively demonstrate that structural investigations into the molecular basis of plant metabolism and regulation provide both answers and inspiration. The combined use of biochemical, biophysical, and computational methods further strengthens the capacity of structural biology to guide rational methods in plant biotechnology. Emerging techniques, such as cryogenic electron tomography, are expected to deliver new breakthroughs. Despite the progress, results relevant to plants remain extremely limited, and we are still far from a complete understanding of the molecular machinery inside plant cells. Significant structural work still lies ahead.

